# Mechanistic investigation into the differences in growth performance and resistance to spring viremia of carp virus in common carp

**DOI:** 10.3389/fimmu.2026.1721974

**Published:** 2026-01-30

**Authors:** Kai Lei, Qiang Li, Jian Zhou, Yong-Qiang Deng, Ping Ouyang, Yang Feng, Sen-Yue Liu, Yan Liu, Hua Ye, Cheng-Yan Mou

**Affiliations:** 1 Key Laboratory of Freshwater Fish Reproduction and Development (Ministry of Education), Key Laboratory of Aquatic Science of Chongqing, College of Fisheries, Southwest University, Chongqing, China; 2 Fisheries Research Institute, Sichuan Academy of Agricultural Sciences, Chengdu, Sichuan, China; 3 Department of Basic Veterinary, College of Veterinary Medicine, Sichuan Agricultural University, Chengdu, Sichuan, China

**Keywords:** association analysis, common carp, disease resistance, growth performance, omic analysis

## Abstract

**Introduction:**

Spring viremia of carp virus (SVCV) is a highly infectious pathogen threatening common carp (*Cyprinus carpio*). Hence, implementing protective measures is crucial to safeguard aquatic species and minimize economic impacts, including pharmaceuticals, vaccines, and breeding of resistant varieties. Common carp is a major aquaculture species, with breeding programs primarily focused on enhancing growth performance. However, evidence indicates that accelerated growth may compromise disease resistance, suggesting a trade-off between these traits.

**Methods:**

We challenged two carp populations with contrasting growth rates using SVCV and performed integrated multi-omics analyses.

**Results:**

Survival analysis showed that fast-growing carp had significantly lower survival than slow-growing carp, with growth negatively correlated with resistance (r = -0.83). RNA-seq analysis of head kidney tissues identified a Grey module gene via Weighted Gene Co-expression Network Analysis (WGCNA), positively linked to growth but negatively to resistance. KEGG enrichment analysis showed that genes within this module were significantly enriched in pathways related to xenobiotic metabolism, nutrient processing, and detoxification. Protein–protein interaction (PPI) network analysis highlighted three hub genes (*gsta4*, *adh8b*, and *gimap7*) potentially regulating both traits. Metabolomic profiling of liver tissues, combined with WGCNA, revealed three metabolite modules: Florawhite, positively associated with body weight and negatively with survival rate; Grey60, negatively associated with body weight and positively with survival rate; and Lightsheelblue, positively associated with survival rate only. These metabolites were predominantly enriched in pathways related to nutrient metabolism, digestion and absorption, and secondary metabolite biosynthesis. Correlation analysis between Grey module genes and metabolites identified 20 genes significantly associated with 159 metabolites. Among these, *ss18*, *arl5a*, and *hnrnph3* emerged as potentially pivotal. The metabolites highly correlated with these three genes were predominantly enriched in pathways related to nutrient metabolism, energy metabolism, detoxification and antioxidation, and immune regulation.

**Discussion:**

In summary, our integrated multi-omics analysis suggests that enhanced nutrient absorption and xenobiotic processing may contribute to superior growth performance in the YB population, while elevated detoxification and antioxidant capacity may underlie stronger disease resistance in the CD population. These findings provide insights into the interplay between growth and immunity in carp and offer biomarkers for balanced breeding strategies.

## 1 Introduction

The common carp, a representative species of the family Cyprinidae within the order Cypriniformes, is one of the most extensively farmed freshwater fish worldwide (1). According to the Food and Agriculture Organization of the United Nations (FAO) *The State of World Fisheries and Aquaculture 2024* (SOFIA 2024), global carp production reached 31,788 thousand tons in 2022. This accounted for 51.7% of all finfish species (2). In China, carp is recognized as one of the major freshwater aquaculture species. Based on the *2025 China Fisheries Statistical Yearbook*, carp production in 2024 was approximately 2,939 thousand tons, ranking fourth among all cultured fish species nationwide. The rapid growth rate and strong environmental adaptability of carp have contributed to its status as one of the most important freshwater aquaculture species globally (3). Over the past decades, selective breeding and hybridization have been widely implemented to improve growth performance in carp (4, 5). However, increasing evidence suggests that fast-growing individuals often exhibit reduced resistance to pathogens and environmental stressors (6, 7). In coho salmon (*Oncorhynchus kisutch*), for instance, fast-growing strains have been shown to allocate fewer resources to immune defense. Molecular evidence further indicates crosstalk between the growth hormone/insulin-like growth factor (GH/IGF) axis and innate immune signaling pathways (8). “Similarly, studies in carp have revealed that fast-growing individuals are enriched in metabolic pathways such as Glycolysis, Nucleotide metabolism, and Riboflavin metabolism. In contrast, slow-growing individuals display higher expression of genes associated with immune-related pathways, including Th17 cell differentiation, NOD-like receptor signaling, C-type lectin receptor signaling, and Autophagy (9). These findings underscore the complexity of balancing growth and immunity in aquaculture breeding programs. Consequently, breeding for immunocompetence has emerged as a pivotal strategy to simultaneously enhance growth performance and disease resistance in genetic improvement initiatives (10).

SVCV, a member of the Rhabdoviridae family, is the causative agent of spring viremia of carp (SVC). This disease is highly contagious and lethal, affecting cyprinid fish. SVC is characterized by acute hemorrhage and septicemia. It has been reported globally and often results in significant mortality and substantial economic losses in aquaculture. Due to its severe impact on fish farming, SVC has been classified as a Category I animal disease in China. It is also listed by the World Organization for Animal Health (WOAH) in the aquatic animal disease catalog. In recent years, increasing attention has been paid to the molecular mechanisms underlying SVCV infection. High-throughput approaches such as transcriptomics and proteomics have revealed that SVCV infection leads to the upregulation of numerous immune- and autophagy-related genes (11–13). In zebrafish embryos, SVCV infection induces interferon (IFN) expression. Overexpression of IFN enhances resistance to SVCV (14, 15). Moreover, overexpression of zebrafish mitochondrial antiviral signaling protein (MAVS) in fish cells significantly promotes IFN production. This, in turn, suppresses SVCV replication (16). Interestingly, splicing variants of zebrafish MAVS can inhibit RIG-I-mediated IFN induction within the RLR signaling pathway (17, 18). In common carp challenged with SVCV, immunoglobulin classes IgM and Ings were markedly upregulated in the head kidney following infection (19). These studies have primarily focused on the virology, epidemiology, diagnostics, and single-gene molecular and immunological responses to SVCV. However, the mechanisms linking host growth traits and disease resistance remain largely unexplored. Elucidating these integrative host-pathogen interactions is essential for advancing genetic improvement strategies and enhancing disease resilience in aquaculture species.

This study investigates the mechanistic basis of variation in SVCV resistance across common carp populations with divergent growth rates. The analysis is conducted from an immunogenetic breeding perspective. By integrating phenotypic assessments with molecular-level analyses, we reveal the potential immunological trade-offs associated with rapid growth. Our findings provide a theoretical foundation for the coordinated improvement of growth performance and disease resistance in aquaculture breeding programs.

## 2 Materials and methods

### 2.1 Fishes and virus

One-year-old common carp for experimentation were obtained from commercial breeding farms in Yibin (YB) and Chengdu (CD), Sichuan Province, China. The SVCV strain used in this study was provided by Professor Ouyang Ping (Sichuan Agricultural University). All procedures followed protocols and ethical guidelines approved by the Sichuan Fisheries Research Institute (Approval No. 20220323001A).

### 2.2 Construction of SVCV infection model

Fish from the YB (106.4 ± 5.82 g) and CD (26.66 ± 0.75 g) populations were randomly allocated into six groups per population, each consisting of 15 individuals. All groups were maintained in sealed aquaria measuring 65 cm × 55 cm × 45 cm. Experimental groups from each population were intraperitoneally injected with 200 μL of SVCV filtrate. Control groups received an equal volume of phosphate-buffered saline (PBS). The water temperature was consistently maintained at 18 ± 0.5 °C. Continuous filtration and daily water exchanges were performed to ensure optimal water quality. Mortalities were promptly removed from the tanks throughout the experiment.

### 2.3 Sample collection

Sampling was performed at four timepoints: prior to infection (0 days post infection, dpi), and at 1, 3, and 5 dpi. At each designated timepoint, five fish were randomly selected from each population. For euthanasia, fish were immersed in a solution of tricaine methanesulfonate (MS-222; Sigma-Aldrich, USA) at a concentration of 350 mg/L. The solution was buffered with an equimolar amount of sodium carbonate. Fish were maintained in the solution until complete cessation of opercular movement was observed. This typically occurred within 10–15 minutes and ensured humane euthanasia. Immediately following euthanasia, fish were dissected using sterile surgical instruments. The following tissues were collected: head kidney, liver, spleen, and trunk kidney. All samples were rapidly frozen in liquid nitrogen and subsequently stored at -80 °C for downstream molecular analyses. Additionally, liver and spleen tissues were immersion-fixed in 4% paraformaldehyde (PFA; Biosharp, Hefei, China) at 4 °C for 24 hours.

### 2.4 Histological analysis

Tissues fixed in 4% PFA were paraffin-embedded, sectioned at 4 µm, stained with hematoxylin and eosin (H&E), and digitized using a pathology scanner (SQS-40R; Shengqiang Technology, Shenzhen, China).

### 2.5 RNA-seq library construction, sequencing

Total RNA was extracted from head kidney tissues of YB and CD common carp populations at 0,1,3, and 5 days post-infection using TRIzol reagent (Invitrogen, USA). RNA integrity and concentration were evaluated using the Agilent 2100 Bioanalyzer (Agilent Technologies, Palo Alto, USA). Polyadenylated mRNA was enriched using magnetic mRNA capture beads and subsequently fragmented by heat treatment. The resulting RNA fragments were reverse-transcribed into cDNA. The cDNA was purified and processed using the Hieff NGS^®^ Ultima Dual-mode mRNA Library Prep Kit. Library preparation steps included end repair, A-tailing, and ligation of Illumina-compatible sequencing adapters. Size selection of ligation products was performed via agarose gel electrophoresis. PCR amplification was then conducted to enrich the final libraries. Sequencing was conducted on the Illumina HiSeq™ 2500 platform (Gene Denovo Biotechnology Co., Guangzhou, China).

### 2.6 Gene annotation

Raw sequencing data were processed using CLC Genomics Workbench (CLC bio, Denmark) to remove adapter sequences. Adapter sequences, low-quality reads (Q score < 20), ambiguous nucleotides, and short reads shorter than 30 bp were removed. This resulted in high-quality clean reads. These clean reads were then aligned to the common carp reference genome (ASM1834038v1) using HISAT2 (version 2.2.1). Transcript assembly was performed with StringTie (version 2.1.7), and gene expression levels were quantified using RSEM (version1.3.3), with transcript abundance expressed as transcripts per million (TPM).

### 2.7 WGCNA analysis

WGCNA was conducted using relevant R packages, including edgeR (version 3.36.0). The analysis assumed a scale-free topology for the gene co-expression network. A gene co-expression similarity matrix and adjacency function were constructed to model network connections. Topological overlap coefficients (TOC) between nodes were calculated to measure network interconnectedness. Hierarchical clustering was then performed to generate a dendrogram. Finally, the relationships between gene significance (GS) and module membership (MM) were assessed. This analysis was used to identify modules with potential biological relevance.

### 2.8 Extraction, quantitative and qualitative analysis of metabolites

Fresh tissue samples (70 mg) were accurately weighed and placed into 2 mL centrifuge tubes. Each sample was mixed with 1,000 μL of pre-chilled methanol (−20 °C), vortexed for 1 minute, and centrifuged at 12,000 rpm for 10 minutes at 4 °C. A total of 450 μL of the resulting supernatant was transferred into a new 2 mL tube and completely dried using a vacuum concentrator. The dried residue was reconstituted in 150 μL of 80% methanol containing 4 ppm 2-chlorophenylalanine (pre-chilled at −20 °C). The solution was then filtered through a 0.22 μm membrane. The filtrate was transferred into LC-MS vials for subsequent analysis.

Quality control (QC) samples were prepared by pooling 20 μL aliquots from each test sample. Metabolite profiling and relative quantification were performed using a liquid chromatography–mass spectrometry (LC-MS) system equipped with an AB SCIEX Triple TOF 6600 mass spectrometer (Shanghai, China). Raw mass spectrometry data were processed using the XCMS package in R, including peak detection, filtering, alignment, normalization, noise reduction, scaling, and imputation of missing values. A quantitative metabolite matrix was generated, and compound identification was carried out using public databases such as HMDB, MassBank, LipidMaps, mzCloud, KEGG, as well as an in-house spectral library.

To minimize systematic bias, LOESS signal correction was applied based on QC samples. Metabolites with a relative standard deviation (RSD) greater than 30% in QC samples were excluded from further analysis. Multivariate statistical analysis was performed using R software. Partial least squares discriminant analysis (PLS-DA) was employed for dimensionality reduction and visualization of metabolic differences among groups. Prior to analysis, data were scaled, and score plots were generated to illustrate group separation. Statistical significance of metabolites was determined based on P-values (<0.05), variable importance in projection (VIP > 1), and fold change (FC) between groups. Metabolites meeting all criteria were considered statistically significant and selected as potential biomarkers.

### 2.9 Combined transcriptomic and metabolomics analysis

An integrated analysis of transcriptomic and metabolomic data was conducted using KEGG pathway mapping to investigate the functional relationships between genes and metabolites. Pearson correlation coefficients were calculated to evaluate the associations between genes and metabolites. Gene–metabolite pairs with an absolute correlation coefficient greater than 0.5 were ranked. The top 200 pairs were selected for network visualization. This approach facilitated the identification of key genes and metabolites occupying central roles within the regulatory network.

### 2.10 Statistical analysis

All numerical data from the experiments were analyzed by calculating the mean and the Standard Error of the Mean (SEM) from three independent replicates. Statistical significance between groups was assessed using one-way analysis of variance (ANOVA). *Post hoc* multiple comparisons were then performed with SPSS software version 26.0 (IBM Corp., Armonk, NY, USA).

## 3 Result

### 3.1 Differences in disease resistance between common carp populations with different growth rates

**Figure 1:**
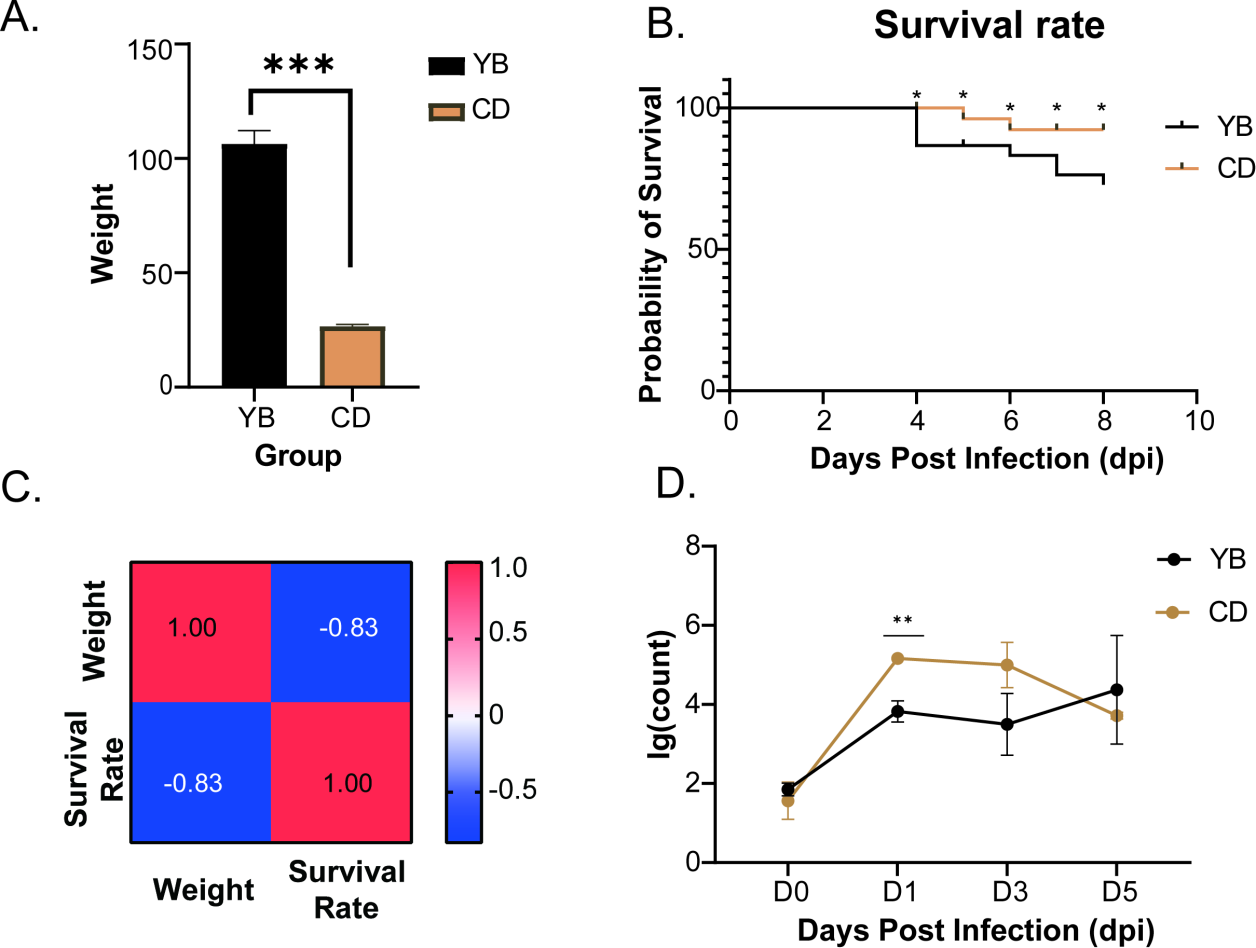
Basic differences between the YB population and the CD population. **(A)** There is an extremely significant difference in weight between the two groups. **(B)** Changes in the survival rates of the two groups over time. **(C)** Heatmap of the correlation between body weight and survival. **(D)** The relationship between the viral copy numbers of the two groups over time. (*p < 0.05, **p < 0.01, ***p < 0.001).

To investigate the relationship between growth and disease resistance, we collected two common carp populations with distinct growth rates and conducted artificial SVCV infection experiments. Fish in the experimental groups showed obvious clinical symptoms, including exophthalmia, abdominal distension, hemorrhages on the skin and fin bases, and a swollen, protruding anus (**Supplementary Figure S1A**). Histopathological analysis of head-kidney tissues revealed autophagy and necrosis in the livers of infected fish, as well as necrosis in their spleens after SVCV infection (**Supplementary Figures S1B, C**). The growth rate of the common carp population from YB was significantly higher than that of the population from CD (*p* < 0.05) (**Figure 1A**). To determine whether growth rate correlates with disease resistance, both populations were infected with SVCV and monitored over eight days. The YB population exhibited a survival rate of 72.8%, whereas the CD population reached 92.3%. The YB population experienced its first mortality on day 4, followed by continuous deaths from days 6 to 8. In contrast, the CD population showed initial mortality on day 5, with deaths occurring only on 5 dpi and 6 dpi (**Figure 1B**). Correlation coefficient analysis indicated a negative correlation between growth and disease resistance in common carp, with a Pearson correlation coefficient of –0.83 (**Figure 1C**). Viral copy numbers in the head-kidney tissues of the two populations were detected at different time points (**Figure 1D**). Apart from the first day post infection, when the viral copy number in the CD population was significantly higher than that in the YB population, no significant differences were observed on other days. Interestingly, the viral copy number in the CD population peaked on the first day of infection and then gradually decreased. In contrast, the viral copy number in the YB population continued to rise. Collectively, these findings indicate that the YB population exhibits superior growth performance but reduced disease resistance.

### 3.2 Transcriptional correlation analysis of growth and disease resistance in common carp populations

**Figure 2:**
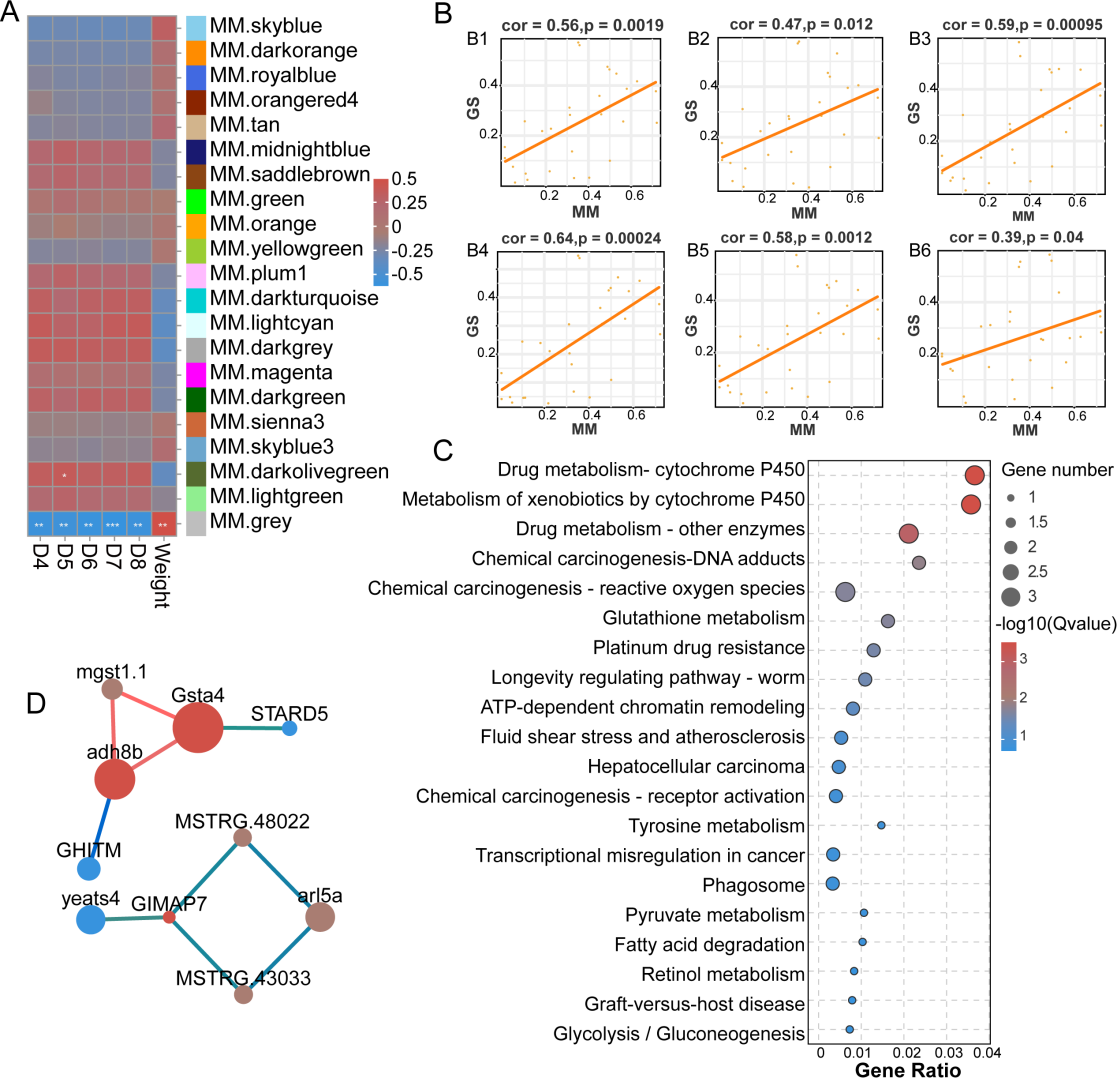
Gene analysis of head kidney transcriptome sequencing in two populations of common carp. **(A)** The heat map of the relationship between each module and the Weight with survival rate through WGCNA analysis. **(B)** MM-GS scatter plots of the Grey module with survival rates (B1-B5: D4-D8) and Weight (B6). **(C)** The KEGG enrichment bubble chart of genes in Grey module. **(D)** PPI network diagram of key genes.

In order to elucidate the antiviral mechanisms underlying the distinct growth rates observed in two common carp populations, we performed transcriptome sequencing of head-kidney tissues. From the full set of expressed genes identified in both populations, a total of 22,175 genes with an FPKM ≥ 5 in at least one sample were selected for subsequent WGCNA (**Supplementary Table S1**; **Figure 2**). Interestingly, we identified a module—designated as the Grey module—that exhibited a significant positive correlation with growth traits, while showing a significant negative correlation with survival rate (**Figures 2A, B**). A total of 28 genes were identified within the Grey module (**Supplementary Table S2**). KEGG enrichment analysis revealed that these genes were involved in several pathways primarily focused on xenobiotics biodegradation and metabolism (Drug metabolism - cytochrome P450, Metabolism of xenobiotics by cytochrome P450, and Drug metabolism - other enzymes), Human Diseases, and other nutrient metabolism (Glutathione metabolism, Tyrosine metabolism, Fatty acid degradation, and Glycolysis/Gluconeogenesis) (**Figure 2C**). The Human Diseases category mainly included pathways associated with cancer. To further reveal the interactions among these genes, we performed a PPI network analysis. This analysis identified three hub genes—*gsta4*, *gimap7*, and *adh8b*—which exhibited the highest degree of connectivity, indicating their central roles within the network (**Figure 2D**). Altogether, these findings suggest that the observed differences in growth performance and disease resistance between the two populations may be primarily attributed to variations in pathways related to xenobiotic metabolism, energy metabolism, cell proliferation, and apoptosis.

### 3.3 Metabolomics correlation analysis of growth and disease resistance in common carp populations

**Figure 3:**
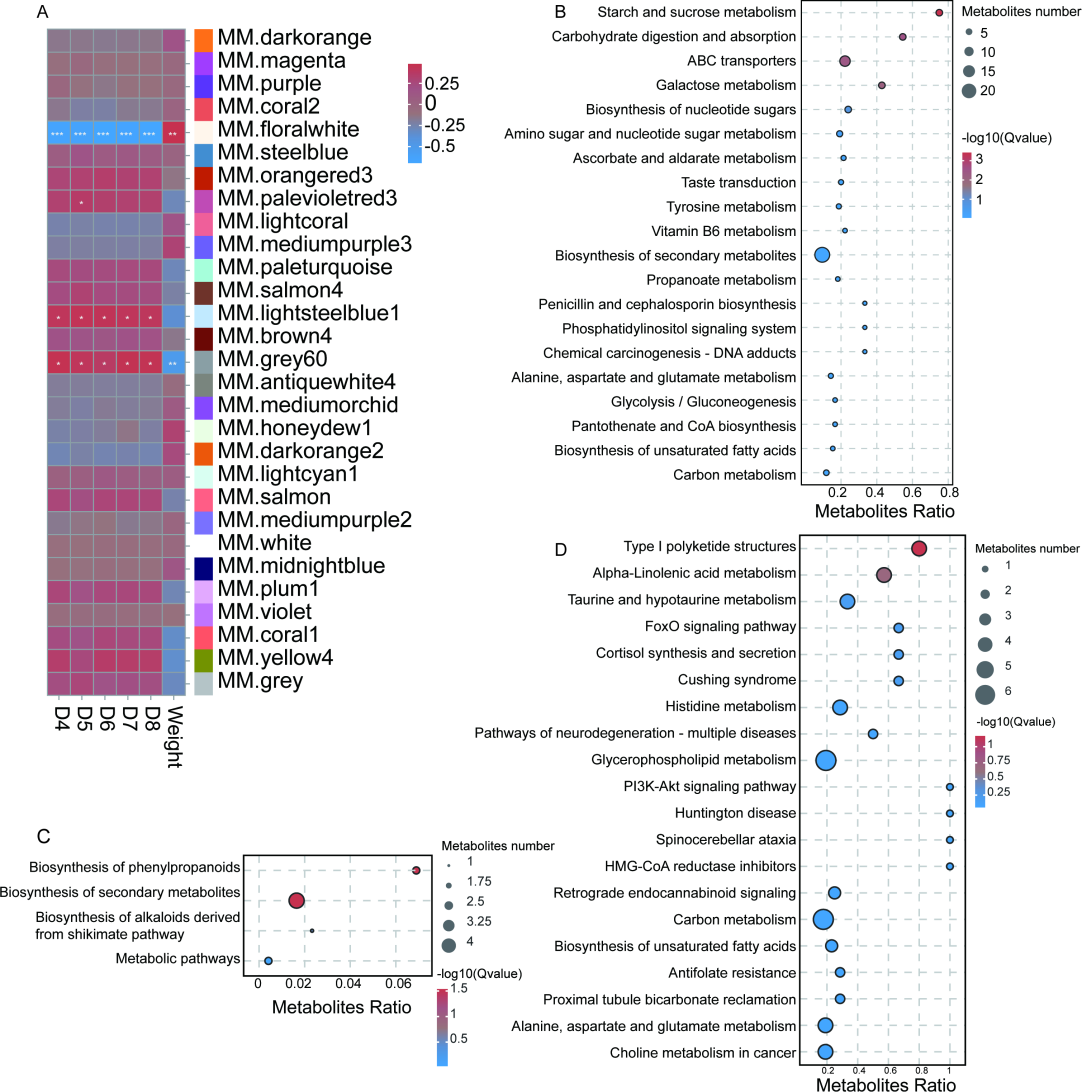
metabolites analysis of liver metabolomic in two populations of common carp. **(A)** The heat map of the relationship between each module and the Weight with survival rate through WGCNA analysis. **(B–D)** The KEGG enrichment bubble chart of metabolites in module. **(B)** Florawhite module. **(C)** Lightsheelblue module. **(D)** Grey60 module.

To further elucidate the antiviral mechanisms underlying the divergent growth rates observed in two common carp populations, we performed metabolomic profiling of liver tissues. From the full set of identified metabolites, 22,682 were selected for WGCNA (**Supplementary Table S3**; **Supplementary Figure S2**; **Figure 3**). Consistent with transcriptomic findings, the Florawhite module showed a highly significant positive correlation with body weight and a significant negative correlation with survival rate (**Figure 3A**). In contrast, the Grey60 module showed a negative correlation with body weight and a positive correlation with survival rate. The Lightsheelblue module showed only a positive correlation with survival rate (**Figure 3A**). A total of 1,686, 185, and 1,856 metabolites were identified in the Florawhite, Lightsheelblue, and Grey60 modules, respectively (**Supplementary Tables S4–S6**). KEGG pathway analysis indicated that the metabolites in the Florawhite module were significantly enriched in four pathways: Starch metabolism and Sucrose metabolism, Galactose metabolism, and ABC transporters (*p* < 0.05) (**Figure 3B**). Metabolites in the Lightsheelblue module showed significant enrichment in two pathways, namely Biosynthesis of phenylpropanoids and Biosynthesis of secondary metabolites (*p* < 0.05) (**Figure 3C**). Similarly, in the Grey60 module, significant enrichment was observed for two pathways: Type I polyketide structures and Alpha-linolenic acid metabolism (*p* < 0.05) (**Figure 3D**). Taken together, these findings suggest that the divergent growth performance and disease resistance between the two common carp populations primarily stem from distinct metabolic strategies. These strategies govern the synthesis, trafficking, and functionalization of key molecules, as well as differences in the organisms’ intrinsic digestive and absorptive capacities.

### 3.4 Association between genes and metabolites

**Figure 4:**
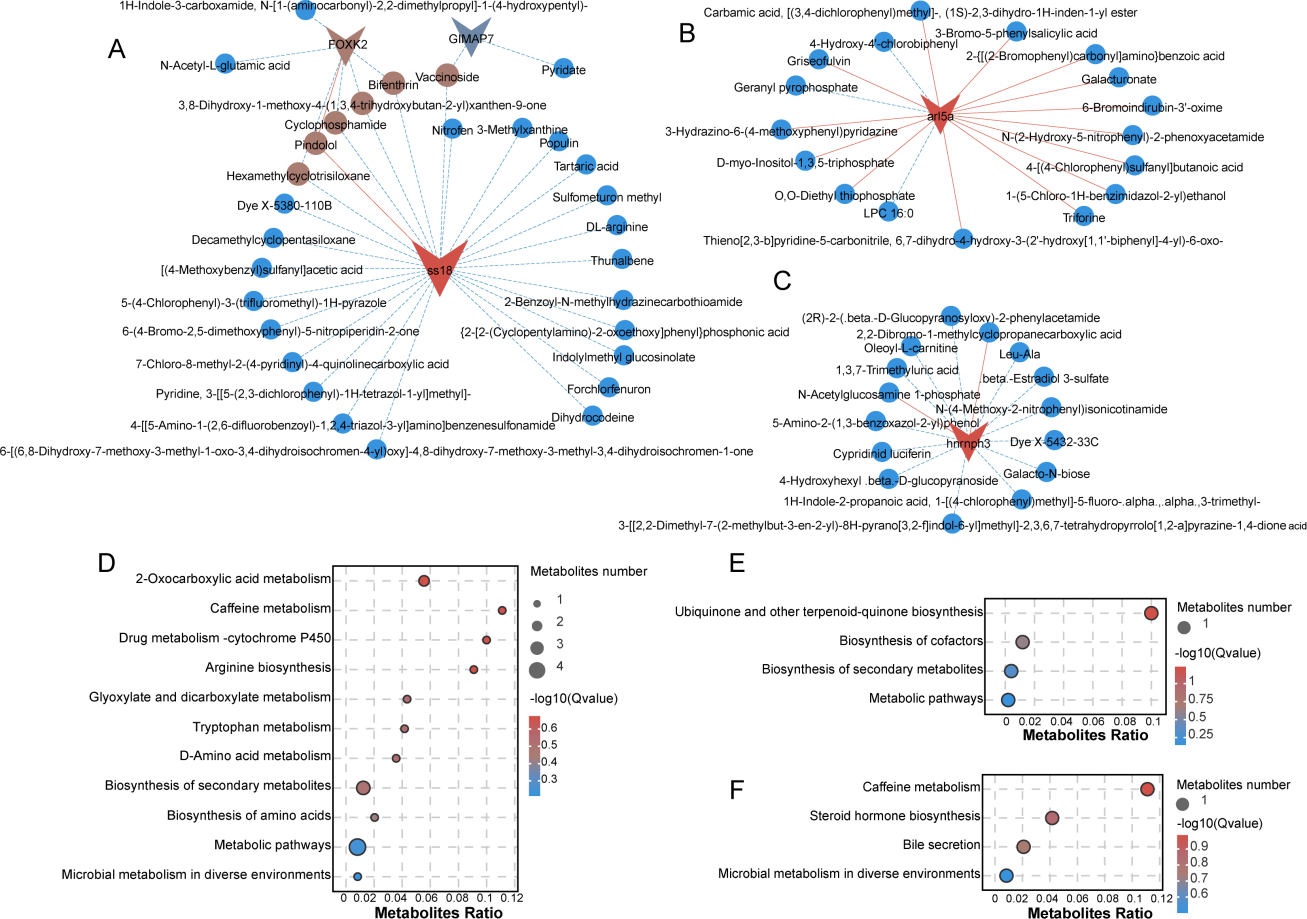
Integrated analysis of Grey module gene–metabolite associations. **(A–C)** Correlation networks between key genes and associated metabolites: **(A)** ss18, foxk2, and gimap7; **(B)** arl5a; **(C)** hnrmph3. **(D–F)** KEGG pathway enrichment of metabolites identified in **(A–C)**, respectively: **(D)** metabolites associated with genes in **(A)**; **(E)** metabolites associated with genes in **(B)**; **(F)** metabolites associated with genes in **(C)**.

To further elucidate how genes interact with metabolites to regulate growth and disease resistance, we performed correlation analysis between the genes in the aforementioned Grey module and all metabolites. As shown in **Figure 4**, the gene with the highest number of associated metabolites is *ss18*, while the metabolites linked to *foxk2* largely overlap with those of *ss18* (**Figure 4A**). Subsequently, *arl5a* (**Figure 4B**) and *hnrnph3* (**Figure 4C**) exhibited the greatest enrichment of metabolites. Other genes, including *thap11*, *cep44*, *mhcII*, *yeats4*, *stard5*, *dipk2ab* and *mgst1.1*, showed intermediate levels of metabolite associations (**Supplementary Figure S3**). In contrast, *prkd4*, *gsta4*, *tc1a*, *nfic*, *mpx*, *ghith*, *gimap7* and Gene *adh8b*, were associated with relatively few metabolites (**Supplementary Figure S3**). These findings suggest that *ss18*, *arl5a*, and *hnrnph3* may play critical roles in balancing growth and disease resistance in common carp. Subsequently, KEGG enrichment analysis was performed on their associated metabolites. The metabolites correlated with *ss18* were significantly enriched in pathways related to essential growth substrates, such as Metabolic pathways, Arginine biosynthesis, and Biosynthesis of amino acids. They were also enriched in energy metabolism pathways, including 2-Oxocarboxylic acid metabolism and Glyoxylate and dicarboxylate metabolism (**Figure 4D**). Additionally, enrichment in Caffeine metabolism, Drug metabolism – cytochrome P450, and Biosynthesis of secondary metabolites suggests a role in detoxification and antioxidant defense. Pathways such as Tryptophan metabolism and D-Amino acid metabolism further imply involvement in immune regulation. These findings indicate that *ss18* may contribute to both growth performance and disease resistance in fish through multifaceted metabolic regulation. The enrichment of *arl5a*-associated metabolites in general biosynthesis pathways—specifically in Ubiquinone and other terpenoid-quinone biosynthesis and Cofactor biosynthesis—underscores their critical roles in both energy metabolism and antioxidant defense (**Figure 4E**). Metabolites correlated with *hnrnph3* were enriched in diverse metabolic processes, including Caffeine metabolism, Steroid hormone biosynthesis, Bile secretion, and Microbial metabolism in diverse environments (**Figure 4F**). This indicates that *hnrnph3* may play a critical role in digestion and nutrient absorption, environmental adaptation, and immune modulation.

## 4 Discussion

It is well documented that different breeds exhibit distinct growth performances (20, 21), a phenomenon that plays a pivotal role in the selective breeding of high-yield aquaculture species. However, populations with enhanced growth traits often display reduced tolerance to environmental stressors (22), a trend consistent with our findings. In this study, we observed that the YB population demonstrated significantly higher growth performance but lower disease resistance compared to the CD population. Body weight is a key indicator of growth performance, with higher weight at the same age reflecting superior growth capacity (23). In our experiment, both populations were one year old, yet the average body weight of the YB population was 106.4 g, while that of the CD population was only 26.7 g-a highly significant difference. Post-SVCV challenge mortality metrics, including time to first death and overall mortality rate, are critical indicators of disease resistance (24). The YB population exhibited earlier onset of mortality—one day ahead of the CD population—and a significantly higher mortality rate. Viral load in tissues is another important measure of disease resistance, as lower viral titers typically indicate stronger antiviral responses and better immunocompetence (25). Additionally, our results showed that the CD population, which exhibited stronger disease resistance, had higher viral loads at 1 and 3 DPI. This phenomenon may be explained by the fact that all fish were injected with the same volume of viral suspension regardless of body weight. Given that CD individuals were significantly smaller than YB individuals, the initial viral dose per gram of body weight was substantially higher in the CD group This factor potentially contributed to the elevated viral load.

Through transcriptomic, metabolomic, and integrative analyses, this study systematically elucidates the molecular mechanisms underlying the divergent growth performance and disease resistance between two common carp populations. Multi-omics results consistently point to two major functional modules driving these phenotypic differences: (1) nutrient and energy metabolism pathways associated with growth performance, (2) detoxification, antioxidant defense, and immune regulation pathways associated with disease resistance.

Rapid growth in fish requires efficient nutrient absorption and energy conversion. Transcriptomic analysis revealed that activation of glycolysis facilitates the utilization of dietary polysaccharides, thereby enhancing feed conversion efficiency (26). Gluconeogenesis complements glycolysis to maintain glucose homeostasis and ensures a stable energy supply (27). Fatty acid degradation via β-oxidation in mitochondria or peroxisomes generates acetyl-CoA. This metabolite enters the tricarboxylic acid (TCA) cycle, which is an essential process for sustained energy production (28). The alcohol dehydrogenase gene Adh8b, highly expressed in the YB population, catalyzes the oxidation of alcohols. It contributes to pyruvate metabolism and fatty acid degradation, increasing acetyl-CoA production and supporting rapid cell proliferation (29). Tyrosine, a semi-essential amino acid derived from phenylalanine, serves as a precursor for growth-related hormones such as adrenaline, noradrenaline, triiodothyronine (T3), and thyroxine (T4) (30). Dietary supplementation with tyrosine has been shown to significantly improve growth rate and weight gain in triploid rainbow trout (*Oncorhynchus mykiss*) *(*31).

Metabolomic analysis further supports these findings. In the Florawhite module, which was positively correlated with growth, pathways related to carbohydrate digestion and absorption—such as starch and sucrose metabolism and galactose metabolism—were significantly enriched. Carbohydrates are a major energy source for fish under aquaculture conditions (32). Efficient Starch metabolism and Sucrose metabolism enhances feed utilization, and forms the basis for rapid growth. Galactose, derived from lactose hydrolysis, is converted into glucose through a series of enzymatic reactions (33). UDP-galactose, an intermediate product, is a key substrate for glycosylation reactions. It contributes to the synthesis of membrane glycoproteins and glycolipids, which are essential for signal recognition and immune function (34). ABC transporters, which rely on ATP hydrolysis, were also enriched. They are known to facilitate transmembrane transport of nutrients and drugs, supporting both nutrient uptake and energy metabolism (35).

Integrative analysis revealed that metabolites associated with ss18 and arl5a were significantly enriched in arginine biosynthesis, biosynthesis of amino acids, 2-oxocarboxylic acid metabolism, and glyoxylate and dicarboxylate metabolism. Amino acids are fundamental building blocks for protein synthesis, which is essential for muscle development and cellular growth. Arginine, an essential amino acid for fish, has been shown to improve feed conversion efficiency and promote growth (36). The 2-oxocarboxylic acid metabolism pathway connects the degradation of multiple amino acids to the TCA cycle. This indicates its dual role in amino acid utilization and energy production. Glyoxylate and Dicarboxylate metabolism pathway, typically active in plants and microorganisms, enables cells to metabolize two-carbon compounds such as ethanol and acetate when monosaccharides are unavailable (37). This pathway can generate glucose from acetyl-CoA, supporting cell growth (38). These findings suggest that the activation of these genes may promote growth performance through coordinated regulation of amino acid synthesis and energy metabolism.

Enhanced disease resistance depends on robust detoxification mechanisms and immune regulation. Transcriptomic analysis identified significant enrichment of xenobiotic metabolism pathways, including drug metabolism via cytochrome P450 and other phase I and II enzymes such as UDP-glucuronosyl transferases (UGTs) and sulfotransferases (SULTs). These enzymes convert exogenous compounds into water-soluble metabolites for excretion (39–41). These pathways collectively protect fish from xenobiotic toxicity and contribute to stress resilience. In fish, xenobiotic metabolism is not limited to the detoxification of pollutants or drugs but also regulates endogenous stress responses triggered by viral infection. Tort and Balasch (42) noted that the fish immune system must remain sensitive to both xenobiotics and pathogens, since pollutants and other exogenous compounds in aquatic environments, together with pathogen exposure, impose stress on the immune system. Sánchez Velázquez (43) further emphasized that under chronic environmental stress, fish activate detoxification and antioxidant systems such as cytochrome P450, glutathione peroxidase, and catalase, which not only participate in xenobiotic metabolism but also modulate oxidative stress and cytokine production, thereby influencing immune responses. Key genes such as *gsta4* and *mgsta1*, members of the glutathione S-transferase (GST) family, were consistently enriched in cancer-related pathways including chemical carcinogenesis, hepatocellular carcinoma, and platinum drug resistance. These enzymes catalyze the conjugation of glutathione with electrophilic compounds. This reaction facilitates detoxification and protects cells during immune activation (44). Activation of the Glutathione metabolism pathway further enhances cellular responses to oxidative stress (45). Beyond cancer, Glutathione metabolism is also a critical pathway during viral infection. It removes reactive oxygen species (ROS) and inflammation-associated molecules, thereby maintaining redox homeostasis and supporting host defense (46). Evidence from influenza virus studies shows that viruses can down−regulate antioxidant systems, such as G6PD activity, to promote oxidative stress and facilitate replication, highlighting the importance of glutathione in antiviral protection (47). More broadly, viral infections are associated with increased ROS and impaired antioxidant responses, which lead to inflammation and tissue damage. This further underscores the central role of glutathione metabolism in mitigating infection-induced stress (48).DNA adducts formed during viral infection can be recognized by the cGAS-STING pathway, triggering type I interferon responses (49). Reactive oxygen species (ROS) not only eliminate pathogens but also activate the RIG-I/MAVS pathway, promoting antiviral immunity (50). Gimap7, predominantly expressed in immune organs and T cells, has been shown to correlate positively with CD4^+^ and CD8^+^ T cell infiltration and immune checkpoint activity, highlighting its immunoregulatory potential (51).

Metabolomic analysis revealed that ABC transporters act synergistically with detoxification enzymes such as CYP450, enhancing the elimination of harmful substances (52). Several secondary metabolite biosynthesis pathways were enriched in modules positively correlated with disease resistance. Type-I polyketide structures, primarily produced by fungi, include bioactive compounds such as antibiotics (e.g., erythromycin) with antimicrobial properties (53, 54). Alpha-linolenic acid (ALA, 18:3n-3), an ω-3 polyunsaturated fatty acid, has demonstrated antiviral effects in grouper (*Epinephelus lanceolatus*) by activating NF-κB and Nrf2 signaling pathways to suppress SGIV infection (55). Phenylpropanoids, synthesized from phenylalanine, possess diverse biological activities including antioxidant, anti-inflammatory, antimicrobial, antidiabetic, neuroprotective, and anticancer effects (56). Secondary metabolites, derived from endogenous synthesis, symbiotic microbes, feed additives, or environmental stimuli, play multifaceted roles in antimicrobial defense, oxidative stress mitigation, and immune enhancement (57–59). Activation of the biosynthesis of secondary metabolites pathway may therefore enhance the adaptability of fish to environmental challenges.

Integrative analysis further highlighted the enrichment of caffeine metabolism, tryptophan metabolism, and D-amino acid metabolism pathways in metabolites associated with ss18, arl5a, and hnrnph3. Caffeine metabolism is linked to purine metabolism and redox regulation, contributing to antioxidant defense. In Nile tilapia (*Oreochromis niloticus*), caffeine supplementation has been shown to reduce oxidative damage and improve hepatic antioxidant enzyme activity under hypoxic stress (60). Tryptophan metabolism plays a critical role in immune regulation. In European seabass (*Dicentrarchus labrax*), dietary tryptophan supplementation enhanced the expression of anti-inflammatory cytokines and modulated macrophage activity during chronic inflammation (61). D-amino acids, primarily produced by bacteria, are metabolized by D-amino acid oxidase (DAO). This enzyme generates hydrogen peroxide (H_2_O_2_), which exhibits antimicrobial activity (62). In common carp, DAO is highly expressed in the intestine, hepatopancreas, and kidney, indicating its role in mucosal defense against exogenous D-amino acids (63). These findings suggest that the activation of these pathways may contribute to enhanced disease resistance through coordinated regulation of detoxification and immune-related metabolic processes.

**Figure 5:**
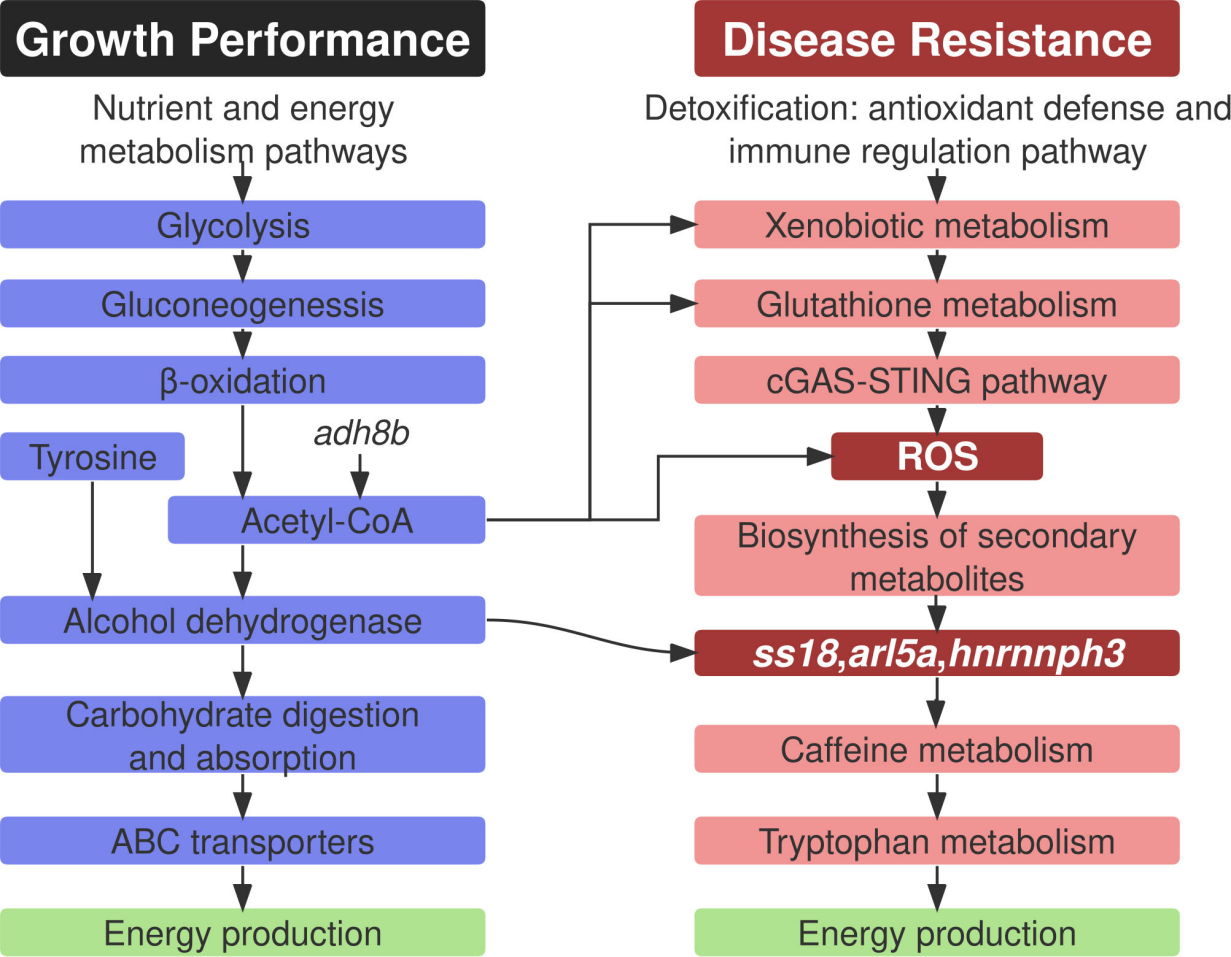
Schematic representation of the molecular mechanisms underlying divergent growth performance and disease resistance in two common carp populations. The diagram is divided into two functional modules: Growth Performance (left) and Disease Resistance (right). Arrows indicate functional interactions and metabolic flow between pathways.

To visualize the integrative multi-omics findings, we constructed a mechanistic diagram summarizing the key metabolic and immune pathways associated with growth performance and disease resistance (**Figure 5**). The left panel illustrates how nutrient absorption and energy metabolism via glycolysis, gluconeogenesis, and fatty acid β-oxidation support rapid growth, with *adh8b* contributing to acetyl-CoA production. The right panel highlights detoxification and immune pathways, in which *ss18*, *arl5a*, and *hnrnph3* regulate secondary metabolite biosynthesis. Central nodes such as ROS and acetyl-CoA bridge metabolic and immune functions, reflecting the coordinated regulation of phenotype-specific traits.

## 5 Conclusion

In summary, the phenotypic divergence in growth performance and disease resistance between the two carp populations is primarily driven by two functional modules: nutrient and energy metabolism, and detoxification, antioxidant defense, and immune regulation. The consistency across transcriptomic, metabolomic, and integrative analyses reinforces the reliability of these findings and underscores the central role of these pathways in shaping phenotypic outcomes. Future studies should explore the interactions among these pathways to provide deeper insights into population optimization and health management in aquaculture.

## Data Availability

The datasets presented in this study can be found in online repositories. The names of the repository/repositories and accession number(s) can be found in the article/**Supplementary Material**.

## References

[1] Noureen A De Marco G Rehman N Jabeen F Cappello T . Ameliorative hematological and histomorphological effects of dietary trigonella foenum-graecum seeds in common carp (cyprinus carpio) exposed to copper oxide nanoparticles. Int J Environ Res Public Health. (2022) 19:13462. doi: 10.3390/ijerph192013462, PMID: 36294038 PMC9603639

[2] FAO . The state of world fisheries and aquaculture 2024. Blue transformation in action. Rome, Italy: FAO (2024). p. 264. doi: 10.4060/cd0683en

[3] Song Y Zhang W Song Y Zhang W . Balancing growth and sustainability in China’s carp aquaculture: Practices, policies, and sustainability pathways. Sustainability. (2025) 17. doi: 10.3390/su17125593

[4] Dong Z Nguyen NH Zhu W . Genetic evaluation of a selective breeding program for common carp cyprinus carpio conducted from 2004 to 2014. BMC Genet. (2015) 16:94. doi: 10.1186/s12863-015-0256-2, PMID: 26219567 PMC4518635

[5] Su S Raouf B He X Cai N Li X Yu J . Genome wide analysis for growth at two growth stages in a new fast-growing common carp strain (cyprinus carpio L.). Sci Rep. (2020) 10:7259. doi: 10.1038/s41598-020-64037-w, PMID: 32350307 PMC7190712

[6] Vandeputte M Gagnaire P-A Allal F . The european sea bass: A key marine fish model in the wild and in aquaculture. Anim Genet. (2019) 50:195–206. doi: 10.1111/age.12779, PMID: 30883830 PMC6593706

[7] Morshedi V Hamedi S Pourkhazaei F Torfi Mozanzadeh M Tamadoni R Ebadi M . Larval rearing and ontogeny of digestive enzyme activities in yellowfin seabream (*acanthopagrus latus*, houttuyn 1782). Comp Biochem Physiol Part A: Mol Integr Physiol. (2021) 261:111044. doi: 10.1016/j.cbpa.2021.111044, PMID: 34371185

[8] Causey DR Kim J-H Stead DA Martin SAM Devlin RH Macqueen DJ . Proteomic comparison of selective breeding and growth hormone transgenesis in fish: Unique pathways to enhanced growth. J Proteomics. (2019) 192:114–24. doi: 10.1016/j.jprot.2018.08.013, PMID: 30153513 PMC7086150

[9] Cai C Yang P Shi Y Wang X Chen G Zhang Q . Transcriptomic and metabolomic analysis revealed potential mechanisms of growth and disease resistance dimorphism in male and female common carp (Cyprinus carpio). Fish Shellfish Immunol. (2025) 158:110150. doi: 10.1016/j.fsi.2025.110150, PMID: 39842680

[10] Liu Z Wu T Xiang G Wang H Wang B Feng Z . Enhancing animal disease resistance, production efficiency, and welfare through precise genome editing. Int J Mol Sci. (2022) 23:7331. doi: 10.3390/ijms23137331, PMID: 35806334 PMC9266401

[11] Yuan J Yang Y Nie H Li L Gu W Lin L . Transcriptome analysis of epithelioma papulosum cyprini cells after SVCV infection. BMC Genomics. (2014) 15:935. doi: 10.1186/1471-2164-15-935, PMID: 25344771 PMC4221675

[12] Liu L Li Q Lin L Wang M Lu Y Wang W . Proteomic analysis of epithelioma papulosum cyprini cells infected with spring viremia of carp virus. Fish Shellfish Immunol. (2013) 35:26–35. doi: 10.1016/j.fsi.2013.03.367, PMID: 23583725

[13] Liu L Zhu B Wu S Lin L Liu G Zhou Y . Spring viraemia of carp virus induces autophagy for necessary viral replication. Cell Microbiol. (2015) 17:595–605. doi: 10.1111/cmi.12387, PMID: 25376386

[14] López-Muñoz A Roca FJ Meseguer J Mulero V . New insights into the evolution of IFNs: Zebrafish group II IFNs induce a rapid and transient expression of IFN-dependent genes and display powerful antiviral Activities1. J Immunol. (2009) 182:3440–9. doi: 10.4049/jimmunol.0802528, PMID: 19265122

[15] Levraud J-P Boudinot P Colin I Benmansour A Peyrieras N Herbomel P . Identification of the zebrafish IFN receptor: Implications for the origin of the vertebrate IFN System12. J Immunol. (2007) 178:4385–94. doi: 10.4049/jimmunol.178.7.4385, PMID: 17371995

[16] Biacchesi S LeBerre M Lamoureux A Louise Y Lauret E Boudinot P . Mitochondrial antiviral signaling protein plays a major role in induction of the fish innate immune response against RNA and DNA viruses. J Virol. (2009) 83:7815–27. doi: 10.1128/JVI.00404-09, PMID: 19474100 PMC2715792

[17] Zhang J Zhang Y-B Wu M Wang B Chen C Gui J-F . Fish MAVS is involved in RLR pathway-mediated IFN response. Fish Shellfish Immunol. (2014) 41:222–30. doi: 10.1016/j.fsi.2014.09.002, PMID: 25219369

[18] Lu L-F Li S Lu X-B Zhang Y-A . Functions of the two zebrafish MAVS variants are opposite in the induction of IFN1 by targeting IRF7. Fish Shellfish Immunol. (2015) 45:574–82. doi: 10.1016/j.fsi.2015.05.019, PMID: 25989622

[19] Wu S Meng K Wu Z Sun R Han G Qin D . Expression analysis of Igs and mucosal immune responses upon SVCV infection in common carp (*cyprinus carpio L.*). Fish Shellfish Immunol Rep. (2022) 3:100048. doi: 10.1016/j.fsirep.2021.100048, PMID: 36419606 PMC9680059

[20] Biro PA . Testing personality-pace-of-life associations via artificial selection: Insights from selected lines of rainbow trout on the context-dependence of correlations. Biol Lett. (2024) 20:20240181. doi: 10.1098/rsbl.2024.0181, PMID: 38949039 PMC11285524

[21] Brezas A Kumar V Overturf K Hardy RW . Dietary amino acid supplementation affects temporal expression of amino acid transporters and metabolic genes in selected and commercial strains of rainbow trout (*oncorhynchus mykiss*). Comp Biochem Physiol Part B: Biochem Mol Biol. (2021) 255:110589. doi: 10.1016/j.cbpb.2021.110589, PMID: 33657457

[22] Dahlke FT Wohlrab S Butzin M Pörtner H-O . Thermal bottlenecks in the life cycle define climate vulnerability of fish. Science. (2020) 369:65–70. doi: 10.1126/science.aaz3658, PMID: 32631888

[23] Yostawonkul J Kitiyodom S Supchukun K Thumrongsiri N Saengkrit N Pinpimai K . Masculinization of red tilapia (oreochromis spp.) using 17α-methyltestosterone-loaded alkyl polyglucosides integrated into nanostructured lipid carriers. Anim (Basel). (2023) 13:1364. doi: 10.3390/ani13081364, PMID: 37106927 PMC10135129

[24] Pasdar Z Pana TA Ewers KD Szlachetka WA Perdomo-Lampignano JA Gamble DT . An ecological study assessing the relationship between public health policies and severity of the COVID-19 pandemic. Healthcare (Basel). (2021) 9:1221. doi: 10.3390/healthcare9091221, PMID: 34574999 PMC8470125

[25] Kandanearatchi A Williams B Everall IP . Assessing the efficacy of highly active antiretroviral therapy in the brain. Brain Pathol. (2003) 13:104–10. doi: 10.1111/j.1750-3639.2003.tb00011.x, PMID: 12580550 PMC8095802

[26] Sari DN Ekasari J Nasrullah H Suprayudi MA Alimuddin A . High carbohydrate increases amylase, plasma glucose, and gene expression related to glycolysis in giant gourami osphronemus goramy. Fish Physiol Biochem. (2022) 48:1495–505. doi: 10.1007/s10695-022-01155-4, PMID: 36454393

[27] Conde-Sieira M Soengas JL . Nutrient sensing systems in fish: Impact on food intake regulation and energy homeostasis. Front Neurosci. (2017) 10:603. doi: 10.3389/fnins.2016.00603, PMID: 28111540 PMC5216673

[28] Betancor MB Ortega A de la Gándara F Tocher DR Mourente G . Performance, feed utilization, and hepatic metabolic response of weaned juvenile atlantic bluefin tuna (thunnus thynnus L.): Effects of dietary lipid level and source. Fish Physiol Biochem. (2019) 45:697–718. doi: 10.1007/s10695-018-0587-9, PMID: 30470945 PMC6500510

[29] Klüver N Ortmann J Paschke H Renner P Ritter AP Scholz S . Transient overexpression of adh8a increases allyl alcohol toxicity in zebrafish embryos. PloS One. (2014) 9:e90619. doi: 10.1371/journal.pone.0090619, PMID: 24594943 PMC3940891

[30] Ravikumar A Deepadevi KV Arun P Manojkumar V Kurup PA . Tryptophan and tyrosine catabolic pattern in neuropsychiatric disorders. Neurol India. (2000) 48:231–8., PMID: 11025626

[31] Zhang S Wang C Liu S Wang Y Lu S Han S . Effect of dietary phenylalanine on growth performance and intestinal health of triploid rainbow trout (oncorhynchus mykiss) in low fishmeal diets. Front Nutr. (2023) 10:1008822. doi: 10.3389/fnut.2023.1008822, PMID: 36960199 PMC10028192

[32] Kamalam BS Medale F Panserat S . Utilisation of dietary carbohydrates in farmed fishes: New insights on influencing factors, biological limitations and future strategies. Aquaculture. (2017) 467:3–27. doi: 10.1016/j.aquaculture.2016.02.007

[33] Ribeiro JPM Mendonça PV Coelho JFJ Matyjaszewski K Serra AC . Glycopolymer brushes by reversible deactivation radical polymerization: Preparation, applications, and future challenges. Polymers (Basel). (2020) 12:1268. doi: 10.3390/polym12061268, PMID: 32492977 PMC7362234

[34] Jumbo-Lucioni P Parkinson W Broadie K . Overelaborated synaptic architecture and reduced synaptomatrix glycosylation in a drosophila classic galactosemia disease model. Dis Models Mech. (2014) 7:1365–78. doi: 10.1242/dmm.017137, PMID: 25326312 PMC4257005

[35] Li A Li J Wang Y Liu Z Liu L Liu L . The metabolomics provides insights into the pacific abalone (haliotis discus hannai) response to low temperature stress. Heliyon. (2024) 10:e40921. doi: 10.1016/j.heliyon.2024.e40921, PMID: 39719996 PMC11666945

[36] Petereit J Lannig G Baßmann B Bock C Buck BH . Circadian rhythm in turbot (scophthalmus maximus): Daily variation of blood metabolites in recirculating aquaculture systems. Metabolomics. (2024) 20:23. doi: 10.1007/s11306-023-02077-9, PMID: 38347335 PMC10861666

[37] Berguson HP Caulfield LW Price MS . Influence of pathogen carbon metabolism on interactions with host immunity. Front Cell Infect Microbiol. (2022) 12:861405. doi: 10.3389/fcimb.2022.861405, PMID: 35372116 PMC8968422

[38] Nishimura Y Watai H Honda T Mihara T Omae K Roux S . Environmental viral genomes shed new light on virus-host interactions in the ocean. mSphere. (2017) 2:e00359–16. doi: 10.1128/mSphere.00359-16, PMID: 28261669 PMC5332604

[39] Djoumbou-Feunang Y Fiamoncini J Gil-de-la-Fuente A Greiner R Manach C Wishart DS . BioTransformer: A comprehensive computational tool for small molecule metabolism prediction and metabolite identification. J Cheminf. (2019) 11:2. doi: 10.1186/s13321-018-0324-5, PMID: 30612223 PMC6689873

[40] Nudischer R Renggli K Hierlemann A Roth AB Bertinetti-Lapatki C . Characterization of a long-term mouse primary liver 3D tissue model recapitulating innate-immune responses and drug-induced liver toxicity. PloS One. (2020) 15:e0235745. doi: 10.1371/journal.pone.0235745, PMID: 32645073 PMC7347206

[41] Alharbi A Alhujaily M . Molecular mechanism of indoor exposure to airborne halogenated flame retardants TCIPP (tris(1,3-dichloro-2-propyl) phosphate) and TCEP tris(2-chloroethyl) phosphate and their hazardous effects on biological systems. Metabolites. (2024) 14:697. doi: 10.3390/metabo14120697, PMID: 39728479 PMC11677016

[42] Tort L Balasch JC . Stress and immunity in fish. In: Buchmann K Secombes CJ , editors. Principles of Fish Immunology : From Cells and Molecules to Host Protection. Springer International Publishing, Cham (2022). p. 609–55. doi: 10.1007/978-3-030-85420-1_20

[43] Sánchez-Velázquez J Peña-Herrejón GA Aguirre-Becerra H Sánchez-Velázquez J Peña-Herrejón GA Aguirre-Becerra H . Fish responses to alternative feeding ingredients under abiotic chronic stress. Animals. (2024) 14. doi: 10.3390/ani14050765, PMID: 38473149 PMC10930682

[44] Jakoby WB . The glutathione S-transferases: A group of multifunctional detoxification proteins. Adv Enzymology Related Areas Mol Biol. (1978) 46:383–414. doi: 10.1002/9780470122914.ch6, PMID: 345769

[45] Sies H Berndt C Jones DP . Oxidative stress. Annu Rev Biochem. (2017) 86:715–48. doi: 10.1146/annurev-biochem-061516-045037, PMID: 28441057

[46] Wróblewska J Wróblewski M Hołyńska-Iwan I Modrzejewska M Nuszkiewicz J Wróblewska W . The role of glutathione in selected viral diseases. Antioxidants. (2023) 12. doi: 10.3390/antiox12071325, PMID: 37507865 PMC10376684

[47] De Angelis M Amatore D Checconi P Zevini A Fraternale A Magnani M . Influenza virus down-modulates G6PD expression and activity to induce oxidative stress and promote its replication. Front Cell Infect Microbiol. (2022) 11:804976. doi: 10.3389/fcimb.2021.804976, PMID: 35071051 PMC8770543

[48] Kayesh MEH Kohara M Tsukiyama-Kohara K . Effects of oxidative stress on viral infections: An overview. NPJ Viruses. (2025) 3:27. doi: 10.1038/s44298-025-00110-3, PMID: 40295852 PMC11993764

[49] Chen Y Yang C Miao Y Shi D Li X Tian S . Macrophage STING signaling promotes fibrosis in benign airway stenosis via an IL6-STAT3 pathway. Nat Commun. (2025) 16:289. doi: 10.1038/s41467-024-55170-5, PMID: 39753529 PMC11698984

[50] Canton M Sánchez-Rodríguez R Spera I Venegas FC Favia M Viola A . Reactive oxygen species in macrophages: Sources and targets. Front Immunol. (2021) 12:734229. doi: 10.3389/fimmu.2021.734229, PMID: 34659222 PMC8515906

[51] Cui L Shen Y Duan S Ding Q Wang Y Yang W . GIMAP7 inhibits epithelial-mesenchymal transition and glycolysis in lung adenocarcinoma cells via regulating the smo/AMPK signaling pathway. Thorac Cancer. (2024) 15:286–98. doi: 10.1111/1759-7714.15150, PMID: 38151913 PMC10834198

[52] Ferreira M Costa J Reis-Henriques MA . ABC transporters in fish species: A review. Front Physiol. (2014) 5:266. doi: 10.3389/fphys.2014.00266, PMID: 25101003 PMC4106011

[53] Wang J Deng Z Liang J Wang Z . Structural enzymology of iterative type I polyketide synthases: Various routes to catalytic programming. Nat Prod Rep. (2023) 40:1498–520. doi: 10.1039/D3NP00015J, PMID: 37581222

[54] Adamantidi T Panoutsopoulou E Stavrakoudi E Tzevelekou P Kokkinos NC . Industrial catalytic production process of erythromycin. Processes. (2024) 12:1533. doi: 10.3390/pr12071533

[55] Liu L Zhang Y Yuan M-D Xiao D-M Xu W-H Zheng Q . Integrated multi-omics analysis reveals liver metabolic reprogramming by fish iridovirus and antiviral function of alpha-linolenic acid. Zool Res. (2024) 45:520–34. doi: 10.24272/j.issn.2095-8137.2024.028, PMID: 38682434 PMC11188608

[56] Zhu Z Chen R Zhang L . Simple phenylpropanoids: Recent advances in biological activities, biosynthetic pathways, and microbial production. Nat Prod Rep. (2024) 41:6–24. doi: 10.1039/D3NP00012E, PMID: 37807808

[57] Almeida JF Marques M Oliveira V Egas C Mil-Homens D Viana R . Marine sponge and octocoral-associated bacteria show versatile secondary metabolite biosynthesis potential and antimicrobial activities against human pathogens. Mar Drugs. (2023) 21:34. doi: 10.3390/md21010034, PMID: 36662207 PMC9860996

[58] Siddik MAB Francis P Rohani MF Azam MS Mock TS Francis DS . Seaweed and seaweed-based functional metabolites as potential modulators of growth, immune and antioxidant responses, and gut microbiota in fish. Antioxidants. (2023) 12:2066. doi: 10.3390/antiox12122066, PMID: 38136186 PMC10740464

[59] Marcharla E Vishnuprasadh A Gnanasekaran L Vinayagam S Sundaram T Ganesan S . The role of functional feed in modulating fish gut microbiome to enhance resistance against aquaculture pathogens. Probiotics Antimicrob Proteins. (2025). doi: 10.1007/s12602-025-10660-w, PMID: 40694305

[60] Baldissera MD Souza CF Descovi SN Petrolli TG da Silva AS Baldisserotto B . A caffeine-supplemented diet modulates oxidative stress markers and prevents oxidative damage in the livers of nile tilapia (oreochromis niloticus) exposed to hypoxia. Fish Physiol Biochem. (2019) 45:1041–9. doi: 10.1007/s10695-019-00616-7, PMID: 30747312

[61] Azeredo R Peixoto D Santos P Duarte I Ricardo A Aragão C . Dietary tryptophan plays a role as an anti-inflammatory agent in european seabass (dicentrarchus labrax) juveniles during chronic inflammation. Biology. (2024) 13:309. doi: 10.3390/biology13050309, PMID: 38785791 PMC11117642

[62] Sasabe J Suzuki M . Emerging role of D-amino acid metabolism in the innate defense. Front Microbiol. (2018) 9:933. doi: 10.3389/fmicb.2018.00933, PMID: 29867842 PMC5954117

[63] Sarower MG Okada S Abe H . Molecular characterization of d-amino acid oxidase from common carp cyprinus carpio and its induction with exogenous free d-alanine. Arch Biochem Biophys. (2003) 420:121–9. doi: 10.1016/j.abb.2003.09.035, PMID: 14622982

