# Supplementary figures and images for: Mechanistic investigation into the differences in growth performance and resistance to spring viremia of carp virus in common carp
Source: Front Immunol. 2026 Jan 30;17:1721974. doi: 10.3389/fimmu.2026.1721974 (PMC12900753; doi:10.3389/fimmu.2026.1721974)

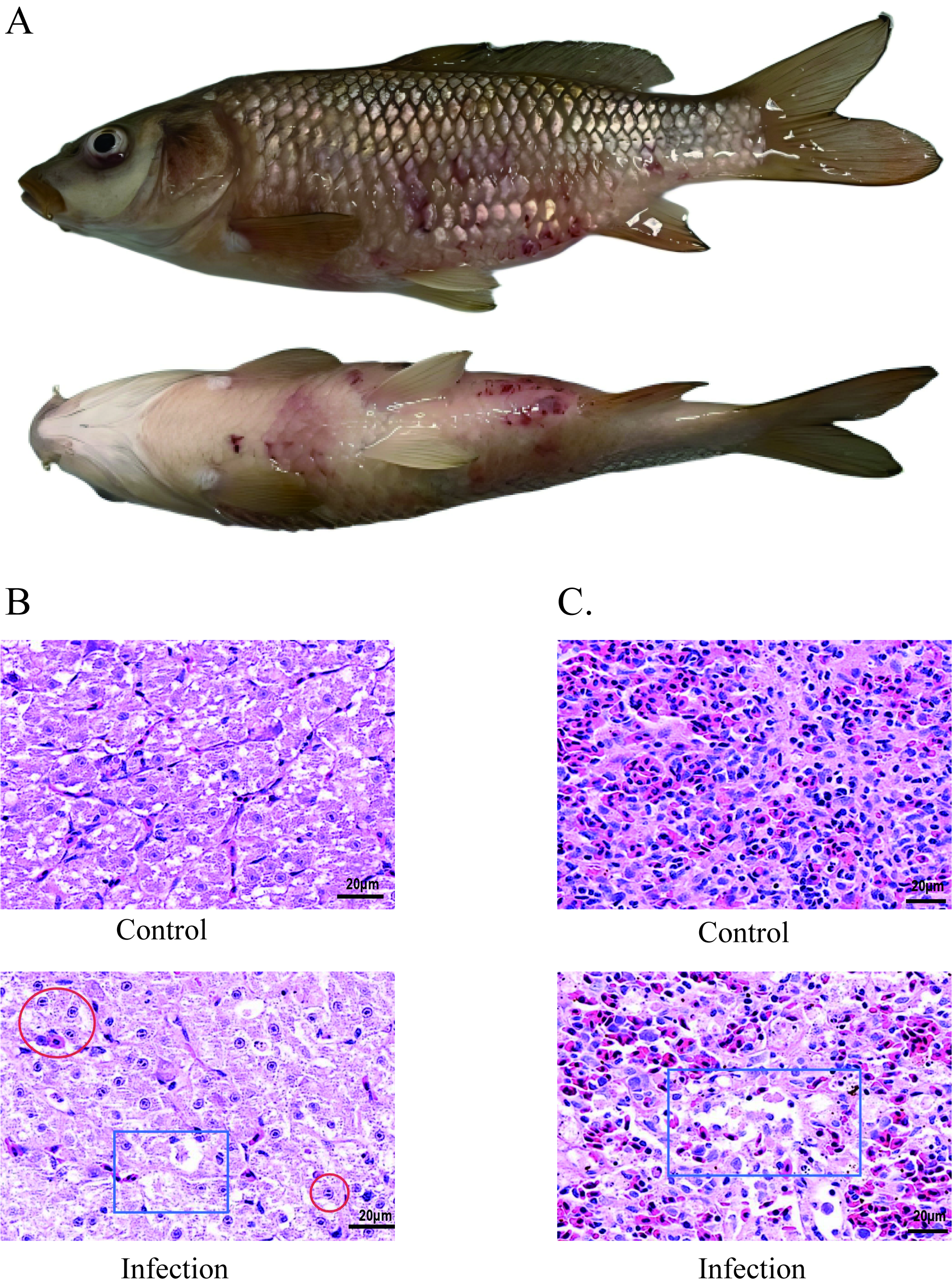

Supplement: Supplementary file 1 [file Image1.tif]

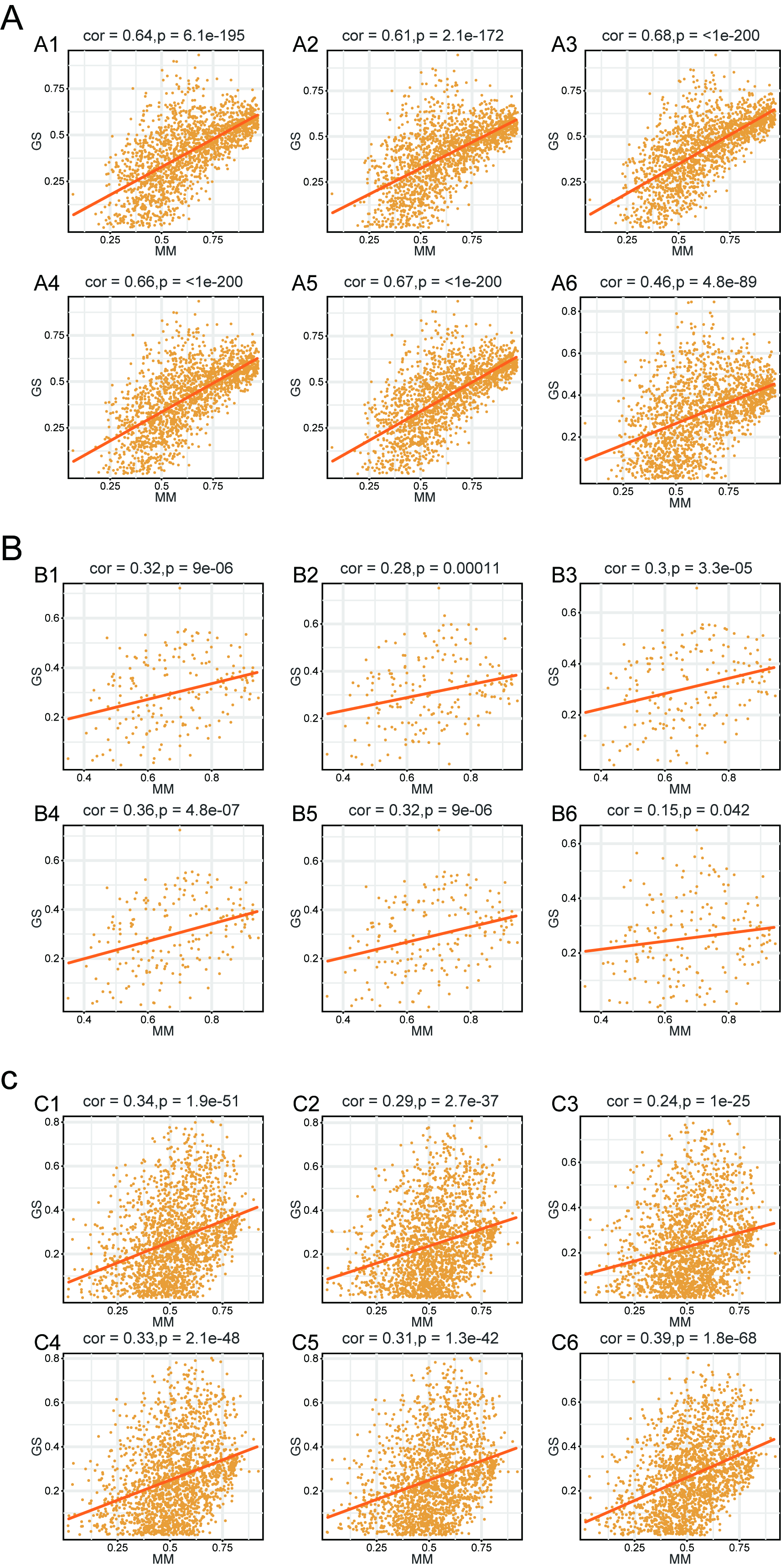

Supplement: Supplementary file 2 [file Image2.tif]

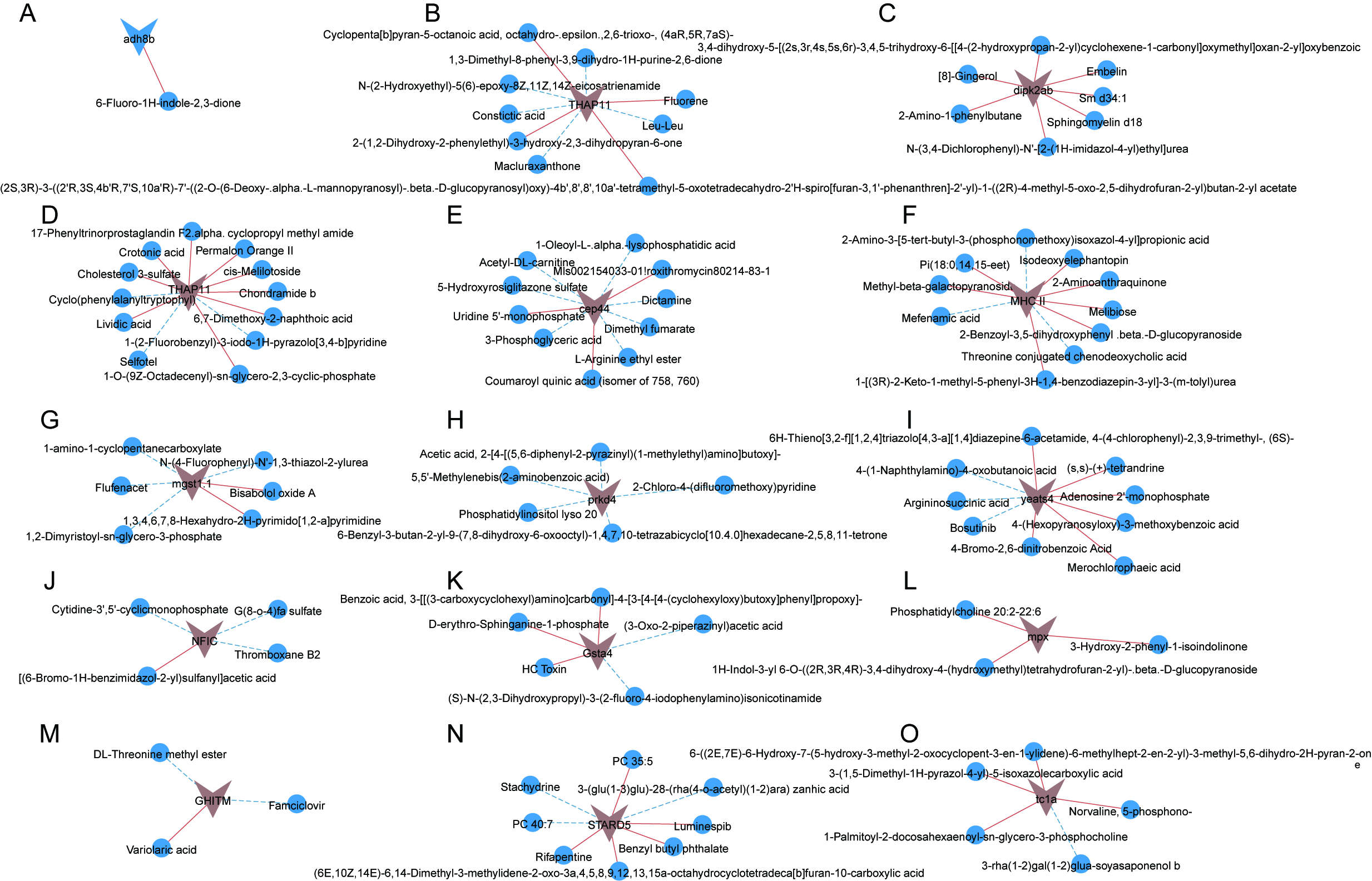

Supplement: Supplementary file 3 [file Image3.tif]
