# Supplementary material for: Mechanistic investigation into the differences in growth performance and resistance to spring viremia of carp virus in common carp
Source: Front Immunol. 2026 Jan 30;17:1721974. doi: 10.3389/fimmu.2026.1721974 (PMC12900753; doi:10.3389/fimmu.2026.1721974)
Supplement: Supplementary file 4 [file Table1.docx]

Fig. S1. Clinical symptoms and histopathological changes in diseased common carp following SVCV infection. (A) clinical signs. (B) Hematoxylin and eosin (HE) staining of liver tissue. (C) HE staining of spleen tissue.

Fig. S2. Correlation between Module Membership (MM) and Gene Significance (GS) in modules based on metabolomic WGCNA analysis. (A) Florawhite module with survival rates (A1-A5: D4-D8) and Weight (A6); (B) Lightsteelblue module with survival rates (B1-B5: D4-D8) and Weight (B6); (C) Grey60 module with survival rates (C1-C5: D4-D8) and Weight (C6).

Fig. S3. Integrated analysis of Grey module gene-metabolite associations. (A-O) Correlation networks between key genes and associated metabolites: (A) adh8b; (B) thap11; (C) dipk2ab; (D) thap11; (E) cep44; (F) mhc Ⅱ; (G) mgst1; (H)prkd4; (I) yeats4; (J) nfic; (K) gsta4; (L) mpx; (M) ghitm; (N) stard5; (O) tc1a.

Table S1. List of genes selected for WGCNA analysis based on predefined filtering criteria.

Table S2. List of genes included in the Grey module identified through WGCNA analysis.

Table S3. List of metabolites for WGCNA analysis.

Table S4. List of metabolites included in the Florawhite module identified through WGCNA analysis.

Table S5. List of metabolites included in the Lightsheelblue module identified through WGCNA analysis.

Table S6. List of metabolites included in the Grey60 module identified through WGCNA analysis.
